# Bi- and trispecific immune cell engagers for immunotherapy of hematological malignancies

**DOI:** 10.1186/s13045-023-01482-w

**Published:** 2023-07-27

**Authors:** Antonio Tapia-Galisteo, Luis Álvarez-Vallina, Laura Sanz

**Affiliations:** 1 Immuno-Oncology and Immunotherapy Group, Biomedical Research Institute Hospital Universitario, 12 de Octubre, Madrid, Spain; 2 Cancer Immunotherapy Unit (UNICA), Department of Immunology, Hospital Universitario, 12 de Octubre, Madrid, Spain; 3 H12O-CNIO Cancer Immunotherapy Clinical Research Unit, Centro Nacional de Investigaciones Oncológicas (CNIO), Madrid, Spain; 4 Molecular Immunology Unit, Biomedical Research Institute Hospital Universitario Puerta de Hierro Majadahonda, Madrid, Spain

**Keywords:** Antibody engineering, Bispecific antibody, Trispecific antibody, T cell engager, NK cell engager, Tumor-associated antigen, Cancer immunotherapy, Hematological malignancy

## Abstract

Immune cell engagers are engineered antibodies with at least one arm binding a tumor-associated antigen and at least another one directed against an activating receptor in immune effector cells: CD3 for recruitment of T cells and CD16a for NK cells. The first T cell engager (the anti-CD19 blinatumomab) was approved by the FDA in 2014, but no other one hit the market until 2022. Now the field is gaining momentum, with three approvals in 2022 and 2023 (as of May): the anti-CD20 × anti-CD3 mosunetuzumab and epcoritamab and the anti-B cell maturation antigen (BCMA) × anti-CD3 teclistamab, and another three molecules in regulatory review. T cell engagers will likely revolutionize the treatment of hematological malignancies in the short term, as they are considerably more potent than conventional monoclonal antibodies recognizing the same tumor antigens. The field is thriving, with a plethora of different formats and targets, and around 100 bispecific T cell engagers more are already in clinical trials. Bispecific NK cell engagers are also in early-stage clinical studies and may offer similar efficacy with milder side effects. Trispecific antibodies (engaging either T cell or NK cell receptors) raise the game even further with a third binding moiety, which allows either the targeting of an additional tumor-associated antigen to increase specificity and avoid immune escape or the targeting of additional costimulatory receptors on the immune cell to improve its effector functions. Altogether, these engineered molecules may change the paradigm of treatment for relapsed or refractory hematological malignancies.

## Background

The outcome of patients with relapsed/refractory (R/R) hematological malignancies has been traditionally poor, but this situation has begun to change in the last years as new and effective agents are being approved for clinical use. The most promising developments include immunotherapeutic strategies such as chimeric antigen receptor (CAR) bearing T cells (CAR-T cell therapy) and bispecific antibodies (BsAbs).

As of 18 July 2023, nine BsAbs are marketed in EU or UE (seven since 2021), another two are under regulatory review and four in late-stage development [1]. Another two were approved in China and Japan in 2022. Except for three, the rest are intended for oncological indications, and nine out of fourteen are T cell engagers (TCE) for the treatment of hematological malignancies: five approved (blinatumomab, mosunetuzumab, teclistamab, epcoritamab and glofitamab), two in review (talquetamab and elranatamab) and two in late-stage trials (odronextamab and linvoseltamab) (Table 1). As an evolution of BsAb, trispecific antibodies (TsAb) have experienced increasing interest in recent years. However, TsAb and natural killer (NK) cell engagers (NKCE) lag behind BsAb, as they entered the clinical arena later, and are in early-stage trials. Globally, more than 100 multispecific antibodies (BsAbs, TsAb and even tetraspecific) are being tested in clinical trials worldwide. This surge of interest in multispecific antibodies has been possible thanks to advances in antibody engineering allowing the expression and purification with high yield of antibody constructs with formats, specificities and effector functions not found in nature.

**Table 1:** Multispecific T cell engagers approved, in review or in late stage clinical trials, as of July 2023

Drug name	Format	Specificity	Indication	Clinical trial	Phase	Sponsor
Blinatumomab	BiTE	CD19/CD3	ALL	NCT02013167	Marketed 2014	Amgen
Mosunetuzumab (RG7828, CD20-TDB)	Hz IgG1-based, Fc -silent, KIH	CD20/CD3	FL	NCT02500407	Marketed 2022	Genentech-Roche
Epcoritamab (GEN3013)	Hz/hu IgG1-based Duobody	CD20/CD3	DLBCL	NCT03625037	Marketed 2023	Genmab-Abbvie
Glofitamab (RO7082859, RG6026)	Hz IgG1-based 2 + 1 CrossMab	CD20/CD3	DLBCL	NCT03075696	Marketed 2023	Roche
Odronextamab (REGN1979)	Hu IgG4-based, VelociBi	CD20/CD3	NHL	NCT03888105	2 (potentially pivotal)	Regeneron
Teclistamab (JNJ 7957)	Hz IgG4 Duobody	BCMA/CD3	MM	NCT04557098	Marketed 2022	Janssen
Elranatamab (PF-06863135)	Hz IgG2, hinge-mutation	BCMA/CD3	MM	NCT05020236	In review	Pfizer
Linvoseltamab (REGN5458)	IgG4 VelociBi	BCMA/CD3	MM	NCT03761108	2 (potentially pivotal)	Regeneron
Talquetamab (JNJ 7564)	Hz IgG4 Duobody	GPRC5D/CD3	MM	NCT04634552	In review	Janssen

Data obtained from: The Antibody Society. Therapeutic monoclonal antibodies approved or in review in the EU or US (Last access: July 2023); www.antibodysociety.org/resources/approved-antibodies; ClinicalTrials.gov (last access: July 2023); and web pages of sponsors (last access: July 2023)

In this review, we will discuss antibody formats, immunologic background, mechanisms of action, relevant clinical and preclinical literature and future perspectives of BsAb and TsAb aimed to redirect T cells and NK cells toward hematological malignancies. A detailed account of the clinical data and side effects would go beyond the scope of this review and we would like to refer the reader to previously published studies [2–6].

## Engineering multispecific antibodies

**Fig. 1:**
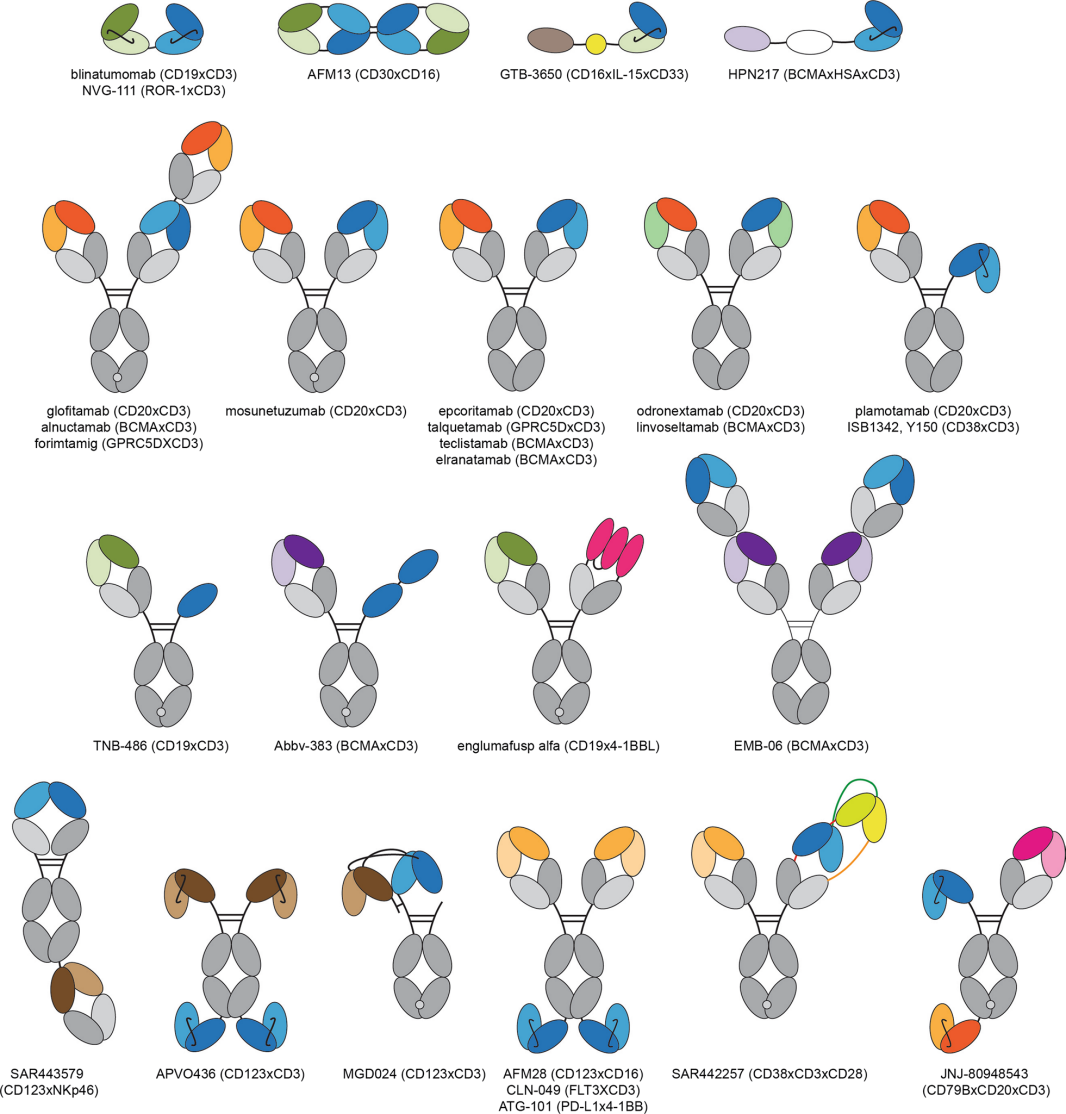
Schematic representation of TCE and NKCE formats approved or in clinical development for hematological malignancies. Five of them are marketed as of July 2023: blinatumomab (Blincyto®), mosunetuzumab (Lunsumio®), teclistamab (Tecvayli®), epcoritamab (Epkinly®) and glofitamab (Columvi®). Additionally, elranatamab and talquetamab are under review, and odronextamab and linvoseltamab are in late-stage clinical trials

A plethora of strategies have emerged to engineer multispecific antibodies, and more than 100 BsAbs [7] and 30 TsAb [8] different formats have been described. In brief, multispecific antibody formats can be classified into two categories: immunoglobulin G (IgG)-like, multichain constructs carrying a fragment crystallizable (Fc) region, and non-IgG-like, Fc-less molecules built upon antibody fragments frequently joined into a single polypeptide chain by flexible linkers. The antibody fragments used as building blocks are: (1) single domain antibodies (sdAb), the variable domain from heavy-chain only antibodies found in camelids (called VHH) or cartilaginous fish (called VNAR); (2) single-chain variable fragments (scFv), comprising the variable regions of heavy (VH) and light (VL) chains joined by a flexible linker; and 3) fragment antigen binding (Fab), which contain the full light chain (VL + CL) and VH and CH1 heavy chain domains, connected by an interchain disulfide bond. Combinations of these fragments have given rise to a variety of multispecific formats (tandem scFv, diabody, tandem diabody, etc.), although these antibody domains can also be appended to the N- or C-terminal ends of standard IgG or IgG-like constructs to endow them with new specificities. The tandem scFv consist of two scFv connected by a flexible linker in a single polypeptide chain; when one of the scFv is directed against a tumor-associated antigen (TAA) and the other against CD3, it is known as “Bispecific T cell Engager” (BiTE). In fact, the first BsAb approved by the Food and Drug Administration (FDA) was a BiTE, the anti-CD19 × anti-CD3 blinatumomab. On the other hand, a bispecific diabody is composed of two different chains, each containing VL and VH domains from two different antibodies, in a head-to-tail arrangement, while the bispecific “Dual Affinity Re-Targeting” (DART) construct is based on a diabody scaffold stabilized by addition of an interdomain disulfide bond, and tandem diabodies (TandAb) are tretravalent antibodies constructed by joining two diabodies with peptide linkers (Fig. 1).

Regarding the multichain IgG-like formats, the main challenge has been the correct pairing of heterodimeric heavy chains between them and with their corresponding light chains [7]. To solve the first problem different strategies have been implemented, being the “Knob into Hole” (KiH) the most widespread technology. Its rationale is to include modifications in CH3 domains to enforce the heterodimerization of two different heavy chains. For light chain association, a frequent solution is to select a common chain for both heavy chains [9]. Several platforms have been developed with different approaches for heavy and light chain pairing, such as CrossMab, Duobody, DVD-Ig (Dual Variable Domain Immunoglobulin), VelociBi, XmAb, ANKET (Antibody-based NK cell Engager Technology) and ADAPTIR, among others.

The presence of an Fc region in IgG-like multispecific antibodies improves overall stability and warrants a long serum half-life [7]; however, their increased size may compromise tumor penetration. On the other hand, non-IgG-like Fc-less formats are smaller molecules with better tissue penetration capacity, but also short-lived in circulation requiring frequent or continuous infusion.

Overall, the multispecific antibody landscape is a blossoming field with a wide variety of formats that can be further classified according to their mechanisms of action.

## Immune cell engagers for redirection of T and NK cells

All BsAb/TsAb approved or in clinical development for hematologic malignancies are immune cell engagers, with at least one of the two (or three) specificities intended to bind and recruit T (for T cell engagers, TCE) or NK cells (for NK cell engagers, NKCE) and another one for binding to a TAA. ICE are molecules capable of redirecting immune effector cells (regardless of their antigen specificity) against cancer cells, acting as a bridge between them to trigger efficient cell killing. Most of TCE contain a binding domain directed against CD3 within the T cell receptor (TCR) complex [1] (Tables 1, 2). Substitution of the anti-CD3 moiety with an anti-CD16 antibody enables recruitment of NK cells (Table 3). In both cases, the addition of a third binding domain endows ICE with new properties, such as increased tumor specificity or enhanced immune-cell activation.

**Table 2:** Multispecific T cell engagers in early stage clinical trials (initiating 2019–2023), as of July 2023

Drug name	Format	Specificity	Indication	Clinical trial	Phase	Sponsor
Plamotamab (XmAb13676)	XmAb Fab-Fc-scFv	CD20/CD3	NHL, CLL	NCT02924402	1	Xencor
GB261	Hz IgG1-based	CD20/CD3	NHL, CLL	NCT04923048	1/2	Genor Biopharma
CM355	NA	CD20/CD3	NHL	NCT05210868	1/2	Beijing InnoCare
JS203	NA	CD20/CD3	NHL	NCT05618327	1	Shanghai Junshi Bioscience
IGM-2323 (imvotamab)	IgM-based 10 + 1	CD20/CD3	NHL	NCT04082936	1/2	IGM Biosciences
TQB2934	IgG1-based	BCMA/CD3	MM	NCT05646758	1	Chia Tai Tianqing Pharma
Abbv-383 (TNB-383B)	Hu IgG4-based	BCMA/CD3	MM	NCT03933735	1/2	AbbVie
Alnuctamab (CC-93269)	Hz 2 + 1^VH−VL±^ CrossMab	BCMA/CD3	MM	NCT03486067	1	Celgene (BMS)
REGN5459	mAb human VelociBi	BCMA/CD3	MM	NCT04083534	1/2	Regeneron
EMB-06	2 + 2 CrossMab FIT-Ig	BCMA/CD3	MM	NCT04735575	1/2	EpimAb Shangai
Forimtamig (RG6324)	Hz 2 + 1VH-VL ± CrossMab	GPRC5D/CD3	MM	NCT04557150	1	Roche
RO7443904, RG6333	1 + 1 IgG1-based	CD19/CD28	NHL	NCT05219513	1	Roche
Englumafusp α (RO7227166, RG6076)	4-1BBL-anti- CD19 IgG1 fusion	CD19/CD137	NHL	NCT04077723	1	Roche
GNR-084	BiMS format	CD19/CD3	B-ALL	NCT04601584	1/2	GENERIUM
TNB-486	Hu IgG4-based	CD19/CD3	NHL	NCT04594642	1	TeneoTwo
CLN-049	Hz IgG1-like (+ 2 scFv), homodimer	FLT3/CD3	AML, MS	NCT05143996	1	Cullinan Oncology
MGD024	DART-Fc	CD123/CD3	AML	NCT05362773	1	MacroGenics
APVO436	ADAPTIR	CD123/CD3	AML, MDS	NCT03647800	1	Aptevo
IGM-2644	IgM-based 10 + 1	CD38/CD3	MM	NCT05908396	1	IGM Biosciences
ISB 1342	BEAT 1.0	CD38/CD3	MM	NCT03309111	1	Ichnos Sciences
Y150	YBODY (scFv-Fab-Fc)	CD38/CD3	MM	NCT05011097	1	Wuhan YZY Biopharma
XmAb18968	Bispecific Fc domains	CD38/CD3	AML, T-ALL, T-LBL	NCT05038644	1	IST
SLAMF7 BATs	Chemically Hetero-conjugated	SLAMF7/CD3	MM	NCT04864522	1	University of Virginia
Cevostamab (RG6160, BFCR4350A)	1 + 1 CrossMab	FCRH5/CD3	MM	NCT03275103	1	Genentech/Roche
				NCT05535244	1/2	
JNJ-75348780	NA	CD22/CD3	NHL, CLL	NCT04540796	1	Janssen
ATG-101	NA	PD-L1/CD137	NHL	NCT04986865	1	Antengene
ONO-4685	NA	PD-1/CD3	T cell lymphoma	NCT05079282	1/2	Ono Pharmaceutical
NVG-111	Tandem scFv	ROR1/CD3	CLL, MCL	NCT04763083	1	NovalGen
SAR442257	CODV-Fab	CD38/CD3/CD28	MM, NHL	NCT04401020	1	Sanofi
HPN217	TriTAC	BCMA/HSA/CD3	MM	NCT04184050	1	Harpoon Therapeutics
ISB 2001	TREAT	BCMA/CD38/CD3	MM	NCT05862012	1	Ichnos Sciences
CMG1A46	TRIAD IgG-like 1:(1 + 1)	CD19/CD20/CD3	NHL, ALL	NCT05348889	1	Chimagen
JNJ-79635322	Undisclosed	Undisclosed	MM	NCT05652335	1	Janssen
JNJ-80948543	IgG1-based Fab-scFv	CD79b/CD20/CD3	NHL, CLL	NCT05424822	1	Jansen
Emfizatamab (GNC-038)	NA	CD3/CD137/PD-L1/CD19	NHL	NCT04606433	1	Sichuan Baili Pharmaceutical
				NCT05623982	1/2	
MP0533	DARPin	CD33/CD123/ CD70/CD3	AML, MDS	NCT05673057	1/2	Molecular Partners

*NA* not available

**Table 3:** Multispecific NK cell engagers in clinical trials (all stages), as of July 2023

Drug name	Format	Specificity	Indication	Clinical trial	Phase	Sponsor
AFM13	TandAb	CD30/CD16a	HL, NHL	NCT04074746	1/2	Affimed
AFM28	IgG-like ROCK	CD123/CD16a	AML	NCT05817058	1	Affimed
LAVA-051	Gammabody	CD1d/NKT TCR	CLL, MM, AML	NCT04887259	1/2	Lava Therapeutics
IPH6101/SAR443579	ANKET	CD123/CD16/NKp46	AML, MDS	NCT05086315	1/2	Sanofi
IPH6401/SAR445514	ANKET	BCMA/CD16/NKp46	R/R MM	NCT05839626	1/2	Sanofi
CC-92328/DF3001	TriNKET	BCMA/CD16/NKG2D	R/R MM	NCT04975399	1	Celgene (BMS)
CC-96191/DF2001	TriNKET	CD33/CD16/KNG2D	R/R AML	NCT04789655	1	Celgene (BMS)

A crucial step in the development of effective BsAb is the selection of the appropriate target. Relevant TAAs in hematologic malignancies include CD19 in acute B cell lymphoblastic leukemia (B-ALL); BCMA, GPRC5D (G Protein–coupled Receptor class C group 5 member D), FCRH5 (Fc receptor homologs 5) and CD38 in multiple myeloma (MM); CD19, CD20 and CD30 in lymphomas; and CD33 and CD123 in acute myeloid leukemia (AML). Notably, only three TAA (CD19, CD20 and BCMA) are the targets of about 65% of the TCE BsAb in Tables 1 and 2. Other factors that may influence cancer cell killing by multispecific antibodies are affinity, epitope location, flexibility and ability to form immunological synapses (IS).

## T cell engagers

Binding simultaneously to the chosen TAA and CD3 leads to the formation of a lytic IS between cancer and T cells and subsequent secretion of cytolytic proteins, triggering serial tumor cell killing even at very low concentrations of the TCE, independently of the TCR specificity and co-stimulatory signals [10]. This polyclonal T cell recruitment only takes place after antibody clustering on TAA-expressing cancer cells, ensuring the tumor-specific T cell activation [11]. Obviously, monovalent CD3 binding is critical to avoid the side effects associated with systemic CD3 cross-linking in the absence of target cancer cells [12, 13]. Another point to be considered is the presence of a functional Fc domain in Ig-like TCE, which may promote cross-linking of Fc gamma receptor (FcγR)-bearing innate immune cells with T cells, also leading to off-target T cell activation or even lysis. Since cytokine release syndrome (CRS), due at least in part to specific (although supraphysiologic) T cell activation, is already challenging the management of TCE-treated patients, it is not surprising that most of IgG-like TCE entering clinical trials have been endowed with an engineered silent Fc and a single CD3-binding domain. Commonly, mutations in the hinge/CH2 interface abrogate Fc-mediated antibody-dependent cell-mediated cytotoxicity (ADCC) and complement-dependent cytotoxicity (CDC) functions, while FcRn binding is not affected, as the FcRn-IgG interface is located at the CH2/CH3 domains of the IgG, ensuring the long half-life enabled by the Fc region [14].

Given that the success of T cell-redirecting strategies might depend on the quality of the IS, how the TCE format influences the organization of the IS should also be taken into consideration. An appropriate format should ensure a good balance between size, affinity/avidity, targeted TAA and epitope localization, in order to enable a high-quality IS [15]*.* The close proximity of cell membranes is critical to induce the assembly of IS, and therefore a TAA with a large extracellular domain may be suboptimal for synapse formation by TCE, especially if the anti-TAA binding domain targets a distal epitope. This “epitope distance effect” on T cell-mediated killing activity has been demonstrated using a panel of BiTE recognizing antigens of different size or different epitopes on the same antigen [16]. Interestingly, the CD20 × CD3 glofitamab induced efficient conjugate formation between T cells and CD20 + cell lines, despite its high molecular weight (194 kDa), suggesting that other TCE features influence the ability to assemble the IS, beyond size and format [17].

Another issue to take into consideration is the role of the immunosuppressive tumor microenvironment, perhaps not so evident in hematologic malignancies as in solid tumors, but still a potential challenge to T-cell-redirecting therapies. In this regard, a work by Nair-Gupta et al. showed that co-culture of bone marrow stromal cells with AML or MM cells prevented the lysis of tumor cells in vitro and in vivo by anti-CD3 × anti-CD123 or anti-CD3 × anti-BCMA Duobodies, respectively [18]. Blocking of protective cell–cell interactions in combination with TCE-mediated redirection reversed stromal-mediated immunosuppression.

### Bispecific T cells engagers

#### CD20-targeting T cells engagers

CD20 is a tetraspanin membrane protein (33–37 kDa) that has been proposed to play a role as a calcium channel, although its involvement in the B cell receptor (BCR) signaling remains contradictory [19]. CD20 is a nearly ideal target, highly expressed on normal B cells (approximately 100,000 molecules per cell) and frequently overexpressed by non-Hodgkin lymphoma (NHL) [20]. In addition, CD20 is present on pre-B cells, but not on hematopoietic stem cells (HSC) or differentiated plasma cells. In fact, the anti-CD20 rituximab was the first monoclonal antibody (mAb) on the market for cancer treatment, approved in 1997 for patients with R/R NHL, which marked a new era for the management of various B cell malignancies. Rituximab was followed by the anti-CD20 radioimmunoconjugate ^90^Y ibritumomab tiuxetan in 2002, and subsequently by two other “naked” anti-CD20 mAb (ofatumumab, 2009 and obinutuzumab, 2013).

With this background of CD20 as a target antigen, it was only a matter of time before a CD20 × CD3 TCE BsAb reached the market. Mosunetuzumab (RG7828, CD20-T cell-Dependent Bispecific antibody [TDB]) is a humanized IgG1-based BsAb, generated using KIH technology, with an aglycosylated, non-functional Fc domain. In non-human primates (NHP), CD20-TDB potently depleted B cells while exhibiting pharmacokinetics (PK) similar to those of conventional mAb [21]. In a pivotal phase II study in patients with R/R follicular lymphoma (FL) who had received two or more previous therapies (NCT02500407), mosunetuzumab as fixed-duration, single-agent treatment induced an overall response rate (ORR) of 80%, with a remarkable rate (60%) of complete and durable remissions at a median follow-up of 18.3 months [22]. Interestingly, mosunetuzumab induced complete response (CR) in 25% of CAR T-pretreated R/R NHL patients, and CAR-T cell expansion was observed after treatment with mosunetuzumab [23]. In addition, the BsAb showed a favorable safety profile, with a low rate of treatment discontinuation. CRS was the most common adverse event (AE) (44% patients), but only two patients (2%) presented grade ≥ 3 CRS [22]. In June 2022, the EMA granted conditional marketing authorization for mosunetuzumab, and in December 2022, the FDA granted accelerated approval for the treatment of adult patients with R/R FL with two or more prior lines of systemic therapies.

Mosunetuzumab was not the only anti-CD20 TCE in the market for long, since epcoritamab (GEN3013) joined it in May and glofitamab in June 2023. A fourth anti-CD20 × anti-CD3 BsAb (odronextamab) may be submitted to regulatory authorities in 2023. Epcoritamab is an Fc-silent, IgG1-based TCE created using DuoBody technology by controlled Fab-arm Exchange (cFAE) [23]. The process involves separate expression of two parental antibodies (avoiding the light chain pairing problem), which are subjected to controlled reducing conditions followed by preferential heterodimer reassembly. In vitro, epcoritamab was more potent killing NHL cells than three different IgG1-like BsAb with single CD3 and CD20 binding regions, and as potent as a BsAb with a single CD3- and two CD20-binding moieties. In NHP, epcoritamab induced long-lasting B cell depletion, which was comparable after subcutaneous (s.c.) and intravenous (i.v.) administration. Peak plasma levels were lower and delayed after s.c. administration, which was associated with a reduced plasma cytokine levels compared to i.v. administration, with similar bioavailability [24]. Results from the diffuse large B cell lymphoma (DLBCL) expansion cohort of a phase I/II trial with 155 patients treated with s.c epcoritamab until disease progression or non-manageable toxicity (NCT03625037), 39% with prior CAR-T cell exposure, revealed an ORR of 63% and CR rate of 39% at a median follow-up of 10.7 months, with 12 months as the median duration of response [25]. At least one CRS event was observed in 49.7% of patients, mostly grade 1 in severity, with only 2,5% of grade 3 (4 patients out of 157).

Glofitamab was developed using the 2:1 CrossMab technology (2 + 1 CrossMab^VH−VL±^) [9], characterized by a silent Fc and three Fab arms enabling monovalent binding to CD3ɛ and bivalent binding to CD20 (derived from obinutuzumab), with the second CD20 Fab fused to the CD3ɛ-binding arm via a flexible linker to enhance functional affinity for CD20 + cells [26, 27]. In Jan 2023, FDA granted priority review to glofitamab for the treatment of R/R DLBCL, based on positive data from a fixed-duration i.v. pivotal phase I/II study (NCT03075696) with heavily pretreated patients (one-third with prior CAR-T cell therapy). Results at a median follow-up of 12.6 months showed an ORR of 52%, and 39% of patients achieved a CR (35% of those receiving previous CAR-T cells), comparing favorably with other approved therapies [28]. The most common AE was CRS (in 63% of the patients), with grade ≥ 3 CRS in 4%. Interestingly, mosunetuzumab was less effective in patients with DLBCL (CR and ORR rates of 19% and 35%, respectively) [29]. Although cross-trial comparisons need to be interpreted with caution, it is tempting to speculate that glofitamab enhanced avidity due to the presence of two anti-CD20 binding domains may contribute to better control of aggressive disease, at the cost of a relatively higher CRS rate [30].

Odronextamab (REGN1979) is an IgG4-based CD20 × CD3 BsAb with mutations to stabilize the hinge region (S228P) and reduce Fc effector functions (E234P, F234V, L235A). The respective anti-CD20 and anti-CD3 heavy chains, together with a common light chain, were co-expressed, and heterodimers were purified exploiting differences in the affinities of the IgG isotypes for protein A (Veloci-Bi platform) [31]. Results from a phase 1 trial with R/R NHL patients who had previously received anti-CD20 mAb (NCT02290951) showed promising antitumor activity [32]. In December 2022, first data from a pivotal phase 2 trial (NCT03888105) evaluating odronextamab in patients with heavily pre-treated R/R DLBCL were presented, with ORR and CR rates of 49.2% and 30.8%, respectively, (82% ORR in R/R FL) with a median follow-up of 17.1 months [33]. Grade 1–2 CRS were observed in 54% of patients, while grade ≥ 3 CRS events occurred in 7% of patients [32]. However, after adoption of a gradual step-up regimen no grade 3 CRS events or above occurred [33]. A phase 1 clinical trial (NCT05685173) has just been launched to study the combination of odronextamab with a BsAb which cross-links CD22 on tumor cells with CD28-expressing T cells (REGN5837). By adding a costimulatory “signal 2”, REGN5837 could enhance the antitumor activity of odronextamab, as reported in preclinical DLBCL studies using human immune system-reconstituted mice [34].

In addition, at least five anti-CD20 TCE are in early-stage clinical trials. Plamotamab (XmAb13676) combines anti-CD20 Fab and anti-CD3 scFv arms (both humanized) in a Fc scaffold engineered to abrogate FcγR binding. XmAb13676 showed potent in vitro, dose-dependent killing of CD20-expressing lymphoma cells along with strong T cell activation and achieved nearly total depletion of B cells in NHP [35]. In a phase 1 study with DLBCL patients (NCT02924402), the ORR was 52.0% (13/25), with a CRR of 24.0% (6/25) [36].

Other CD20 × CD3 BsAb in phase 1 or 1/2 clinical trials include GB261, IGM-2323, JS203 and CM355. To generate GB261, the heavy chain CDRs in one arm of a rituximab analog were replaced with those from an anti-CD3 mAb. While most TCE in development are Fc-silent (or Fc-free), GB261 exhibits a functional Fc region. The anti-CD3 arm was modified to decrease its affinity, in order to prevent non-specific T cell activation, or their lysis by ADCC, in the absence of CD20 + cells [37]. IgM-2323 (imvotamab) is a bulky molecule with ten high affinity IgG CD20-binding domains grafted on a pentameric IgM framework and a single anti-CD3 scFv fused to the J chain [38]. IGM-2323 high avidity elicited potent in vitro T cell dependent cytotoxicity, even on rituximab-resistant cells with low cell surface expression of CD20, along with CDC. In NHP repeated-dose studies, durable depletion of B cells was observed without significant AE and only with a limited and transient increase of IL6 levels at 4–8 h post-administration. A phase I dose-escalation study of IGM-2323 as i.v. monotherapy is currently ongoing in patients with R/R NHL (NCT04082936), with some evidence of clinical activity [39, 40].

#### CD19-targeting T cells engagers

CD19 is a 95 kDa type I transmembrane glycoprotein of the immunoglobulin superfamily, uniformly expressed on the majority of malignant (and normal) B cells, with a role in their proliferation and survival. CD19 is not only expressed in NHL and in B cell chronic lymphocytic leukemia (B-CLL) like CD20 and CD22, but also in B-ALL.

The CD19 × CD3 BiTE blinatumomab was the first BsAb ever approved in 2014 by the FDA, independently of indication, format or mechanism of action. A 2008 seminal work in Science by Bargou et al. [10] demonstrated tumor regression in NHL patients using very low doses of blinatumomab. Breakthrough designation and accelerated approval in the US were granted based upon data from a pivotal phase 2 trial in Ph^−^ adult R/R B-ALL, with a 43% CR/CRh rate [41]. Subsequent trials in Ph^+^ [42] and pediatric B-ALL [43] demonstrated CR/CRh rates of 36% and 39%, respectively, and the FDA approved these indications in 2017. Importantly, blinatumomab was also approved for the treatment of the minimal residual disease (MRD) with 10^−3^ or greater, a key indicator of event-free survival for all subsets of ALL. The approval by FDA in 2018 was supported by a phase 2 trial in adult B-ALL (NCT01207388), in which 88% of patients reached complete MRD [44, 45]. Very recently, a phase II study demonstrated that the addition of a single cycle of blinatumomab after standard chemotherapy in infants with *KMT2A*-rearranged B-ALL increased two-year disease-free survival from 49.4 to 81.6% [46]. Interestingly, single cells analysis has identified transcriptional changes in immune subpopulations after blinatumomab treatment, shedding light on the potential mechanisms associated to response [47].

Probably the main drawback of blinatumomab is its relatively small size (55 kDa), which added to the lack of a Fc domain results in a short half-life of approximately 2 h in humans, precluding bolus administration and requiring continuous i.v. infusion. However, this can also be an advantage, as blinatumomab infusion may be discontinued in response to adverse effects. Although blinatumomab is a first-generation BiTE with both scFv of murine origin, immunogenicity showed a low incidence across studies, with less than 2% of patients generating anti-drug antibodies (ADA) [48]. This characteristic is shared with all B cell depleting TCE which prevent B cell differentiation and antibody production, but the risk of immunogenicity should be considered when the target cell is different, even if the TCE is humanized, since antibody engineering may give rise to neoantigens [48].

Despite the promising early results of blinatumomab supporting the TCE approach for NHL, the emergence of anti-CD19‐directed CAR-T cells, with four different products approved by the FDA, may have discouraged subsequent BsAb development. Currently, only three anti-CD19 BsAb are in early phase clinical trials, including TNB-486, RG6333 and RG6076.

TNB-486 is a fully human, asymmetric CD19 × CD3 BsAb generated by pairing an anti-CD19 sdAb V_H_ with a low-affinity CD3-binding Fab in a silenced IgG4 backbone using KiH technology [49]. Given its higher molecular weight and the presence of a Fc domain, TNB-486 is expected to have longer half-life than BiTE and therefore a more favorable dosing schedule. In vitro, TNB-486 induced lower cytokine release compared to its high-affinity CD3 binding counterpart. Interim results from the current Phase 1 study of TNB-486 (NCT04594642) showed a tolerable safety profile and promising activity [50]. Apparently, the efficacy of the therapy was not affected by the low affinity of the anti-CD3 moiety, since the ORR and CR were of 72% and 61%, respectively. Even 2 of 3 patients previously treated with CAR-T therapy achieved CR. Notably, a phase I trial (NCT02106091) in patients with R/R NHL treated with another CD19 × CD3 BsAb, whose affinity for CD3 was 100-fold higher than that of blinatumomab, was discontinued due to a non-favorable risk/benefit profile [51].

According to the two-signal model, CD3 engagement must be followed by co-stimulatory signals for optimal T cell activation and proliferation. RG6333 is a heterodimeric 1 + 1 IgG1-based CD19-targeted BsAb containing a low affinity, monovalent anti-CD28 binding moiety and a silent Fc-domain aiming to provide “signal 2” to activated T cells [52]. The clinical use of CD28 agonists is associated to the risk of severe side effects due to cytokine release since the TeGenero clinical trial [53]. On the contrary, RG6333 is designed to provide CD19-targeted co-stimulation to T cells activated by a TCE, with no activity as single agent. Currently, RG6333 is in a phase I, open-label, dose-escalation study with NHL patients (NCT05219513) in combination with the CD20 × CD3 TCE glofitamab.

Another widely characterized co-stimulatory molecule is 4-1BB (CD137) [54]. Englumafusp alfa (RO7227166, RG6076) is not a *bona fide* BsAb, but an anti-CD19 moiety fused to 4-1BB ligand promoting CD19-specific 4-1BB cross-linking on the surface of T and NK cells. Interestingly, alternation of RG6333 with RG6076 completely prevented tumor relapse during glofitamab treatment in vivo [52]. Currently, englumafusp alfa it is in a phase I clinical trial with NHL patients in combination with obinutuzumab or glofitamab (NCT04077723).

#### BCMA-targeting T cells engagers

BCMA (CD269), the major target for BsAb in MM, is a member of the tumor necrosis factor receptor (TNFR) family which promotes cell proliferation and survival after binding its ligands APRIL (A Proliferation Inducing Ligand) and BAFF (B cell Activating Factor). BCMA (14.6 kDa) is expressed at high levels by malignant and normal plasma cells but is absent in HSC and most non-hematological tissues, making it an ideal target for T cell-redirecting strategies. Increased levels of the soluble form of BCMA (sBCMA) have been correlated with disease progression. FDA-approved, BCMA-targeted agents include the CAR-T products idecabtagene vicleucel (2021) and ciltacabtagene autoleucel (2022), as well as the antibody drug conjugated (ADC) belantamab mafodotin, a humanized, afucosylated anti-BCMA IgG1 conjugated to auristatin F, approved in 2020 and withdrawn from the market in 2022, after the confirmatory phase III trial failed to meet FDA Accelerated Approval requirements.

On October 2022, s.c. teclistamab became the first anti-BCMA × anti-CD3 TCE BsAb approved for patients with R/R MM who have failed four prior lines of therapy, including a proteasome inhibitor, an immunomodulatory agent, and an anti-CD38 antibody. Teclistamab is an IgG4 generated from the OmniAb transgenic mouse using DuoBody technology, with an Fc region carrying the S228P stabilizing mutation to prevent Fab-arm exchange and L234A/L235A (PAA) mutations to silence effector functions. In preclinical studies, teclistamab promoted efficient cytotoxicity of MM cells in vitro, even in the presence of sBCMA, and tumor regression in vivo [55]. Its accelerated approval was granted based upon the promising results of a pivotal phase 1/2 multicohort study in 165 patients with R/R MM (NCT03145181, NCT04557098), which showed an ORR of 63% and CR rate of 39.4% [56, 57]. The most common AE was CRS (all grade 1 or 2 events, except for one grade 3 event). Although response ratio was slightly lower than those in CAR-T-treated patients in previous clinical trials (67% for idecabtagene vicleucel y 83% for ciltacabtagene autoleucel), the greater safety profile based on lower CRS and neurotoxicity, along with the easier and faster manufacture, make teclistamab an alternative to other BCMA-targeting therapies [57].

Another anti-BCMA TCE may join teclistamab in the market soon. Elranatamab (PF-06863135, PF-3135) is a humanized IgG2 with anti-BCMA and anti-CD3 arms joined using hinge-mutation technology [58]. On February 2023, the FDA granted Priority Review for elranatamab Biologics License Application (BLA), based mainly on the results of an ongoing phase 2 study (NCT04649359) in 123 patients without prior BCMA-targeted treatment who received s.c. elranatamab, which achieved an ORR of 61% with no grade ≥ 3 CRS [59].

A third BCMA-targeted therapeutic advanced in clinical trials is linvoseltamab (REGN5458), a BCMA × CD3 BsAb generated using VelocImmune technology and VelociBiTM platform. VelocImmune mice, which are humanized with human variable region gene segments [60], were immunized with CD3 or BCMA, and the selected antibodies were assembled into a human IgG4 constant region containing the stabilizing S228P mutation in the hinge region, along with mutations to minimize antibody-mediated effector functions and to limit protein A binding to the anti-CD3 Fc. In vitro, linvoseltamab showed cytotoxic effect on MM cells equivalent to that of CAR-T cells harboring the same anti-BCMA scFv. In vivo, the antitumor potency of both strategies was also similar, although the BsAb eliminated MM with faster kinetics than CAR-T cells [61]. In a phase 1/2 clinical trial (NCT03761108) with 58 MM patients, linvoseltamab induced 64% ORR [62]. REGN5459 is another BCMA × CD3 TCE in earlier clinical trials whose affinity for CD3 has been reduced to mitigate side effects while preserving tumor killing. In a phase 1/2 clinical trial to assess the antitumor activity and safety in R/R MM patients (NCT04083534), the ORR was 65%, with 32.6% classified as sCR. However, CRS risk was apparently similar to that of linvoseltamab [63].

As of the writing of this review, there are at least other four BCMA × CD3 TCE BsAb in early-phase clinical trials: alnuctamab, Abbv-383, EMB-06 and TQB2934. Alnuctamab (BMS-986349; CC-93269) is an asymmetric IgG1-like human antibody based on 2:1 T cell bispecific antibody (TCB) platform, facilitating correct Fc heterodimerization by KiH technology and Fab pairing by VH-VL crossover of the anti-CD3 Fab. This antibody binds bivalently BCMA with high affinity, but monovalent CD3 binding exhibits low affinity in order to minimize side effects. In addition, the Fc region has been engineered with L234A, P329G and L235A mutations to impair ADCC and CDC. Alnuctamab induced tumor regression in 66% of mice in a MM xenograft model and depletion of BCMA^+^ cells NHP [64]. In a phase 1 trial with R/R MM patients treated with i.v. alnuctamab (NCT03486067), 39% achieved an objective response although treatment was associated with a high rate of CRS, including a grade 5 event [65]. Preliminary data for s.c. administration of alnuctamab to improve tolerability showed a decrease in CRS severity, without affecting the antitumor activity in comparison with the i.v. administration (ORR = 51%) [66].

Based on a similar format, Abbv-383 (formerly TNB-383B) is another fully human IgG4-like anti-BCMA × anti-CD3 BsAb engaging BCMA bivalently with high affinity but monovalently with low affinity to CD3 to potentially mitigate the incidence of CRS and eliminate the need for step-up dosing. In a preclinical in vivo study, TNB-383B induced tumor regression in MM-bearing NSG mice [67]. In an early phase 1/2 with R/R MM patients (NCT03933735), TNB-383B demonstrated a 57% of ORR in all patients treated, with the same percentage of CRS [68].

#### Other targets for MM

TCE BsAb against targets alternative to BCMA may constitute an interesting therapeutic option for MM patients after exposure to anti-BCMA CAR-T cells. GPRC5D is a relatively novel target for MM. Of unknown function, GPRC5D (39 kDa) is highly expressed on MM cells, as well as in hard keratinized structures, with low expression in normal tissues. High expression of GPRC5D has been associated with poor prognosis in MM. Interestingly, BCMA and GPRC5D are differentially expressed in MM [69]. Currently, two anti-GPRC5D TCE BsAb, talquetamab and forimtamig, are being tested in clinical trials. In addition, a GPRC5D-targeted CAR-T cell therapy (BMS-986393) is in phase 1.

Talquetamab, a humanized IgG4-based DuoBody with PAA mutations generated by cFAE, is the most advanced in clinical development (NCT03399799/NCT04634552). In preclinical studies, talquetamab efficiently promoted T cell-mediated killing of MM patient samples and induced regression of MM xenografts [70, 71]. In heavily pretreated R/R MM patients, the two s.c. doses recommended for a phase 2 study showed remarkable antitumor activity [72–74]. More than 32% of patients at each dose level had a CR, and combined data showed an ORR of about 73%. Specific AE, concomitant to GPRC5D expression pattern, were dysgeusia, skin-related AEs and nail disorders (primarily low-grade). In Dec 2022, a BLA was submitted to the FDA for talquetamab.

Forimtamig (RG6234) is another GPRC5D-specific TCE BsAb with a 2:1 configuration, similar to glofitamab (CD20 × CD3) and alnuctamab (BCMA × CD3). An ongoing phase I study (NCT04557150) is investigating the i.v. and s.c. administration of RG6234 in patients with R/R MM [75, 76]. ORR were at 71% in the i.v. cohort and 64% in the s.c. cohort.

FcRH5 (CD307) is a 120 kDa type I membrane protein with a large extracellular domain (106.4 kDa) that is expressed exclusively in the B cell lineage, and at a higher level on MM cells than on normal B cells. The FcRH5 gene is located in the chromosome region 1q21, and 1q21 gain could lead to FcRH5 overexpression in patients with high-risk MM. Cevostamab (RG6160) is a FcRH5 × CD3 TCE which binds to a membrane-proximal epitope of FcRH5, leading to efficient synapse formation and MM cell killing [77]. Occupancy calculations indicated that as few as ∼ 50 TCE molecules were sufficient to induce T cell activation and target cell lysis. In an ongoing phase 1 study (NCT03275103), cevostamab was administered i.v. for a fixed duration of 17 cycles, with a ORR = 54.5% at the higher dose level [78] and durable responses [79].

CD38 is a 46 kDa type II transmembrane glycoprotein that is expressed on lymphoid and myeloid cells and on non-hematopoietic tissues. Notably, CD38 is highly expressed on MM cells. CD38 has been found to have multiple functions, including ectoenzymatic activity as well as receptor-mediated regulation of cell adhesion and signal transduction. Daratumumab was the first-in-class human IgG1 anti-CD38 mAb approved for the treatment of MM (2015), followed by isatuximab in 2020. At least three anti-CD38 targeted TCE BsAb are in early phase clinical trials. ISB 1342 was built on the BEAT (Bispecific Engagement by Antibodies based on the TCR) platform, engineered with a scFv arm binding CD3 and a Fab arm which recognizes a CD38 epitope different from that of daratumumab and constructed with a double LALA mutation. In vitro*,* ISB 1342 killed a range of CD38 + cell lines with superior efficacy than daratumumab, including low-expressing CD38 cells that were daratumumab-resistant [80]. A phase 1 clinical trial with R/R MM is accruing additional dose cohorts (NCT03309111) [81]. The Y150 TCE, developed using the YBODY platform [82], is also an asymmetric IgG-like BsAb with scFv-Fab-Fc structure, with the heterodimeric Fc stabilized by KiH and displaying reduced affinity of the anti-CD3 scFv to potentially improve safety (NCT05011097). XmAb968 is another CD38 × CD3 TCE with optimized relative affinities for both CD3 and CD38, currently in a phase 1 trial recruiting patients with T cell acute lymphoblastic leukemia (T-ALL) (NCT05038644) [83], since CD38 expression in leukemic blasts of T-ALL has been found to be robust [84]. IGM-2644 is another IgM-based TCE, with 10 binding sites for human CD38, and a single anti-CD3 scFv. Preclinical data suggest improved safety profile compared to other CD38xCD3 TCE BsAb due to lower cytokine release and reduced T cell fratricide [85]. IGM-2644 entered a phase I clinical trial in June 2023 for R/R MM (NCT05908396).

Most MM cells (and normal plasma cells) also express high levels of Signaling Lymphocytic Activation Molecule Family 7 (SLAMF7, CS1, CD319), a cell-surface receptor that belongs to the signaling-lymphocytic-activation-molecule (SLAM) family, with either low or no expression in normal tissue. In 2015, elotuzumab was the first anti-SLAMF7 mAb approved for the treatment of R/R MM. Currently, a phase I clinical trial is testing the combination of an anti-CD3 × anti-SLAMF7 TCE with peripheral blood mononuclear cells for patients with R/R MM (NCT04864522).

CD33 mediates the proliferation and differentiation of normal and malignant myeloid cells. The approval of the ADC gemtuzumab ozogamicin in 2000 for AML treatment (withdrawn in 2010 and reapproved in 2017) validated CD33 as a therapeutic target. Although different anti-CD33 TCE BsAb entered clinical trials, currently none of them are active. Phase 1 studies with AMG 330 (a CD33 × CD3 canonical BiTE) and AMG 673 (a half-life extended -HLE- BiTE) were terminated for prioritization decision. Other phase 1 trials with JNJ-67571244 and TandAb AMV 564 were completed, with no results posted.

#### T cells engagers for AML

CD123, the IL-3 receptor alpha chain (70 kDa), is expressed at high levels on leukemic blasts and relatively absent on normal HSC. Of note, tagraxofusp, a fusion protein consisting of the CD123 ligand, interleukin 3, linked to a truncated diphtheria toxin payload has become the standard of care for blastic plasmacytoid dendritic cell neoplasm (BPDCN) [86]. MGD024 is a second-generation, CD123 × CD3 DART aimed for the treatment of AML and other hematological malignancies, with reduced affinity for CD3 to ameliorate CRS. This HLE- DART permits intermittent i.v. dosing thanks to the incorporation of an IgG1 Fc region and subsequent increase in half-life [87] compared with the first-generation DART flotetuzumab (MGD006), a continuous-infusion TCE that showed preliminary single-agent activity in AML [88]. A phase 1 study (NCT05362773) of MG024 as monotherapy in patients with CD123 + R/R hematological malignancies was initiated in 2022 [89]. APVO436 is another anti-CD123 TCE developed on the ADAPTIR platform [90], currently in phase 1 clinical trials in patients with AML and myelodysplastic syndrome (MDS) (NCT03647800) [91].

The FLT3 (CD135) mutation status of newly diagnosed AML patients has become crucial in the management of AML. FLT3 is a receptor tyrosine kinase expressed in both AML and B-ALL, with low expression on myeloid dendritic cells and HSCs, which functions as a proto-oncogene playing a key role in promoting leukemic cell proliferation and survival, and expression is preserved in AML after relapse. CLN-049 is a symmetric TCE BsAb which binds the membrane proximal domain of FLT3 independently of the mutational status, constituted by an IgG1-based, Fc-silenced, anti-FLT3 antibody with anti-CD3 scFv fused to the C-termini of both heavy chains [92]. Designed to bind FLT3 with both arms and CD3 monovalently, CLN-049 efficiently promoted lysis of cell lines in vitro with a range of FLT3 expression, and was highly active in vivo in a human AML mouse model [93]. CLN-049 is currently in a phase 1 clinical trial (NCT05143996) for the treatment of patients with R/R AML or MDS.

### Trispecific T cells engagers

Trispecific TCE have at least one of the three binding domains recognizing T cells through CD3, and at least another TAA-targeting moiety. The presence of a third arm endows these constructs with unprecedented properties.

#### Dual targeting of T cell receptors

It is a widely accepted dogma in immunology that TCR/CD3 triggering needs of co-stimulatory signals for optimal T cell activation and proliferation (“signal 2”). Furthermore, activated T cells, in turn, express inhibitory checkpoint receptors to modulate duration and amplitude of the immune response. Hence, the generation of trispecific TCE including agonists of co-stimulatory molecules (such as CD28 or 4-1BB/CD137) or antagonists of checkpoint receptors (such as PD-1), along with the anti-CD3 and anti-TAA binding domains, constitutes a sensible strategy.

In 2020, the study by Wu et al. [94] describing a CD38 × CD3 × CD28 TsAb (named SAR442257) brought this type of TsAb into the spotlight [95]. SAR442257 was generated using the Cross-Over Dual Variable (CODV) antibody technology on a non-complement fixing IgG4 scaffold with the previously described mutations F234A L235A to abrogate FcγR binding. The presence of the anti-CD28 domain in the TsAb enhanced T cell activation and killing of different MM cell lines in vitro, with superior potency compared with the anti-CD38 mAb daratumumab. Of note, this construct (renamed as CD38 TriAb) has shown significantly higher anti-MM cytotoxicity in ex vivo studies with primary MM samples and autologous T cells of patients relapsed after anti-CD38 and anti-BCMA immunotherapies compared to daratumumab and isatuximab [96]. Importantly, maximum MM cell killing in vitro was observed at CD38 TriAb concentrations far below serum levels well tolerated in NHP, precluding the side effects associated with potent CD28 stimulation in vivo. The authors argue that monovalent CD28 binding by the TsAb is less prone to induce cytokine release than the bivalent anti-CD28 IgG used in the TeGenero trial. CD38 TriAb is currently in a phase I clinical trial with MM and NHL patients (NCT04401020), which will ultimately clarify whether safety concerns are warranted in this case [97]*.*

An ideal TCE would comprise not only TAA and CD3 targeting domains and co-stimulatory agonists, but also immune checkpoint blockers, all in one. Such a molecule exists, emfizatamab (GNC-038), an octavalent tetraspecific CD3 × CD137 × PD-L1 × CD19 antibody. Different versions of GNC-038 were obtained, replacing each binding domain with an irrelevant one, and the contribution of each moiety was demonstrated in cytotoxicity assays with CD19 + cells in vitro [98]. The first of its class tested in humans, emfizatamab is in phase 1 or 1/2 trials with R/R NHL patients (NCT04606433, NCT05623982). Appealing in concept, this huge Fc-based antibody may rise concerns about increased risk of CRS, along with unspecific activation due to the presence of two anti-CD3 binding domains.

#### Dual targeting of tumor-associated antigens

Dual TAA-targeted TsAb may prevent tumor escape by antigen loss caused by selective pressure, in comparison with conventional single TAA-targeting TCE BsAb and may help to overcome pre-existing clonal heterogeneity in tumor cells. Dual antigen recognition on B-NHL cells with a trispecific T cell-redirecting antibody has the potential to enhance tumor binding through avidity effects, maximize tumor eradication in the presence of a heterogeneous cell population, and prevent resistance via TAA escape. Approximately 30% of blinatumomab-treated relapses are characterized by the loss of the CD19 antigen [99], rendering malignant cells invisible to CD19-targeted therapies. Similarly, CD20 expression was downregulated in over 20% of DLBCL patients that had relapsed/progressed after R-CHOP [100]. Indeed, a bispecific anti-CD19/CD20 CAR-T product has been designed to address the issue of antigen escape, demonstrating strong efficacy (90% ORR, 70% CR rate) in a first-in-human, phase I dose-escalation trial with NHL patients [101].

To avoid CD19 or CD20 loss when single-targeted, CMG1A46 was designed as an IgG-like CD19 × CD20 × CD3 TsAb based on the TRIAD platform. In vitro, CMG1A46 was able to induce potent lysis of cells expressing both CD19 and CD20, as well as single-positive cells; in vivo, the TsAb demonstrated superior potency compared to a CD3 × CD20 BsAb [100]. A phase I/II clinical trial is undergoing to evaluate the safety and efficacy of CMG1A46 in adult patients with advanced NHL or B-ALL (NCT05348889).

JNJ-80948543 is a fully human IgG1-based TsAb composed of an anti-CD79b Fab and two scFv against CD20 and CD3, along with a silent Fc region. CD79b (previously known as Igβ) is a 26 kDa BCR-associated protein, targeted by the ADC polatuzumab vedotin*,* approved in 2019 for the treatment of R/R DLBCL. JNJ-80948543 was selected based on its high affinity for CD79b and CD20 and low affinity for CD3. Interestingly, JNJ-80948543 mediated cytotoxicity of single TAA-expressing tumor cells but showed > 1000-fold increased potency toward double-positive cells, consistent with an avidity effect. In vivo, this TsAb prevented the growth and induced dose-dependent regression of DLBCL xenografts [102]. Currently, JNJ-80948543 is in a phase 1 trial with NHL and B-CLL patients, administered as s.c. injection (NCT05424822).

Simultaneous targeting of BCMA and CD38 is an appealing strategy for MM treatment. ISB 2001 is a BCMA × CD38 × CD3 TsAb based on the TREAT (Trispecific Engagement by Antibodies based on the TCR) technology [103], consisting of three Fab derived from a synthetic phage display library with common light chains, and a silenced Fc region. Compared to anti-BCMA or anti-CD38 TCE, the binding of ISB 2001 to MM cells with low levels of both TAA was strongly increased due to avidity, and MM cell killing was superior to the combination. On July 2023, ISB 2001 was granted orphan drug designation by the FDA for the treatment of MM, and a phase 1 first-in-human dose-escalation dose-expansion study will be initiated in brief (NCT05862012).

An alternative to Ab-based constructs is the use of non-immunoglobulin scaffolds such as anticalins, affibodies and DARPins. DARPins are based on naturally occurring ankyrin repeat domains, and constitute a scaffold amenable to the generation of multispecific proteins [104]. MP0533 is a tetraspecific TCE DARPin targeting CD33, CD123 and CD70 in addition to CD3, currently in a phase 1/2 clinical trial (NCT05673057) with AML and MDS patients [105].

#### TCE engineered for half-life extension

A third subtype of trispecific TCE comprises the mandatory anti-TAA and anti-CD3 binding domains, along with a moiety for increased serum half-life. TsAb based on small Ab fragments, such as scFv and sdAb, usually exhibit molecular weights below the renal filtration cut-off, despite their multimeric nature. Half-life extension of antibody fragments by fusion to a human serum albumin (HSA)-binding sdAb offers the advantage of more even drug concentration and less frequent dosing. For example, the single-chain TriTAC (trispecific T cell activating construct) format is composed of a humanized anti-TAA sdAb and anti-CD3 scFv, separated by an anti-albumin sdAb, which provides TriTAC with extended serum half-life. Currently, the TriTAC HPN217 (BCMA × HSA × CD3) is in phase 1 clinical trials for treatment of MM patients (NCT04184050). PK studies across dose levels revealed a remarkable half-life of 66 h (for a molecule of 53 kDa), and treatment was well tolerated (only grade 1–2 CRS events), resulting in durable responses [106].

## NK cells engagers

Redirecting NK cells to kill tumors constitutes a potential alternative to T cell-based therapies, which are undoubtedly effective, but at the cost of frequent side effects, the most severe probably CRS. The clinical response observed with anti-CD19 CAR-NK cells, in the absence of major toxic effects [107], has illustrated the potential of harnessing NK cells for cancer immunotherapy [108]. For reasons that are not fully understood, CRS, if it occurs during NK cell therapy, tends to be much milder [109].

Most NKCE (BsAb or TsAb) display an antibody fragment directed against CD16a, the equivalent to the CD3-targeting moiety of TCE. CD16a (FcγRIIIa) is an activating, low-affinity IgG receptor mainly expressed on NK cells and macrophages [110]. In fact, the effector functions of conventional, “naked” mAb directed against TAA (mostly IgG1), such as rituximab, depend mostly in ADCC and antibody-dependent cellular phagocytosis (ADCP) triggered by CD16a binding to Fc domains, along with CDC. ADCC relies on the formation of an IS and the degranulation of lytic granules containing perforin and granzymes, being the killing machinery comparable to that of effector CD8 T cells [111]. Interestingly, there are potential advantages of using an anti-CD16a moiety over the native Fc domain in multispecific constructs, including high affinity binding to both CD16a allotypes 158 V (with higher affinity to Fc) and 158F, no detectable binding to primary human granulocytes (which express CD16b), and conserved NK cell binding in the presence of competing IgG.

### Bispecific NK cells engagers

Based on the BiTE concept, a similar strategy was developed for the generation of NKCE BsAb, named “Bispecific Killer cell Engagers” (BiKE), consisting of two scFv linked through a linker, one of them directed against CD16a and another one targeting a TAA [112]. No BiKE entered clinical trials, despite encouraging preclinical data, but other NKCE BsAb (and TriKE) did (Table 3).

AFM13 is a chimeric TandAb with a murine anti-CD30 binding domain and a human anti-CD16a domain [113]. The anti-CD16a moiety recognizes an epitope which is mostly unaffected by plasma IgG competition and CD16a polymorphism and binds with high affinity binding to its target inducing potent ADCC. AFM13 is the most advanced candidate based on the Redirected Optimized Cell Killing (ROCK) antibody platform which comprises a plethora of CD16a-binding NKCE formats [114]. AFM13 was studied in a phase I trial with patients with R/R CD30 + Hodgkin lymphoma (HL) with limited efficacy as monotherapy, likely due to the impaired function of autologous NK cells in these patients [115]. For this reason, the combination of AFM13 and allogenic NK cells to render a CAR-like NK product may be an alternative approach to treat CD30 + hematologic malignancies [116]. A phase I/II trial (NCT04074746) is currently studying the safety and activity of AFM13-NK administration, followed by AFM13 monotherapy. Preliminary results are promising, with an ORR of 97% and CR of 63% [116]. RO7297089/AFM26 is another TandAb directed against BCMA whose efficacy as single agent in a phase 1 trial with R/R MM patients (NCT04434469) was not as solid as previously reported with BCMA-targeted TCE [117].

AFM28 is a tetravalent anti-CD123 NKCE BsAb which also belongs to the ROCK platform, with a totally different format: a Fc-silent mAb directed against CD123 with scFv anti-CD16 appended at the C-terminal end of both heavy chains. In vitro results in a panel of AML cell lines showed effective ADCC against CD123^+^ cells by allogeneic NK cells and independent blocking of IL-3R signaling [118]. A phase I clinical trial has just been launched in patients with AML (NCT05817058).

Recently, it has been described a BsAb which comprises two VHH anti-CD1d and anti-Vδ2-TCR linked in a single polypeptide chain, able to engage innate-like T cell subsets such as Vγ9Vδ2-T cells along with type 1 NKT cells [119, 120]. This construct, named LAVA-051, demonstrated increased survival in AML, MM, and T-ALL mouse models and good tolerability in NHP. LAVA-051 is currently in a Phase 1–2 clinical trial (NCT04887259) for the treatment of patients with AML, CLL and MM.

### Trispecific NK cells engagers

As discussed above for TCE, NKCE can benefit from improved properties by adding an extra binding domain. For example, dual targeting of AML cells has been pursued with a CD33 × CD16a × CD123 TsAb, which induced significantly stronger NK lysis of primary leukemic cells than the BsAb version, although the optimized candidate SPM-2 did not reach clinical trials [121].

#### Dual targeting of NK cell activation receptors

Similarly to T cells, full activation of NK cells requires the co-engagement of different cell-surface receptors in addition to CD16a. In fact, the activating Natural Killer receptors p30 (NKp30) and p46 (NKp46), as well as Natural Killer Group 2 member D (NKG2D), have gained increasing attention as potential targets for NK cell redirection [122]. Based on this consideration, a series of trispecific NKCE which simultaneously bind a TAA and trigger two NK cell activating receptors have been designed. Some of them have been generated based on ANKET platform. In a pioneering work, Gauthier et al. [123] reported the characterization of different NKCE composed of two Fab antibody fragments targeting NKp46 and a TAA (CD19, CD20 or EGFR), separated by an Fc domain to promote ADCC via CD16. These trifunctional molecules were effective against several tumors in vitro, with no off-target cytotoxicity. In a lymphoma model, the anti-CD20 ANKET controlled disease better than the Fc-silenced counterpart or rituximab. Currently, a phase I clinical study with a CD123 × CD16 × NKp46 ANKET (IPH6101/SAR443579) [124] is ongoing in patients with AML (NCT05086315). SAR443579 is a trifunctional NKCE targeting CD123 on cancer cells and co-engaging NKp46 and CD16a on NK cells, designed to promote the formation of a synapse between NK cells and CD123-positive tumor cells leading to NK cell activation and tumor cell killing. Preclinical studies showed that SAR443579 triggered potent antitumor activity against primary AML blasts and AML cell lines and promoted survival in vivo in an aggressive human AML xenograft model. Treatment of NHP with SAR443579 resulted in sustained depletion of circulating CD123 + cells with minimal cytokine release as compared to an anti-CD123 TCE [125]. On May 2023, a BCMA-targeting ANKET named IPH6401/SAR445514 has entered a phase 1/2 study (NCT05839626) as monotherapy in patients with R/R MM and R/R light chain amyloidosis. Improved NK cell activation had been reported with dual CD16a/NKp46 engagement compared to single CD16a or NKp46 targeting agents or combination of these molecules [126], and cytotoxic activity was only induced in the presence of MM cells and associated with low cytokine release.

Still in preclinical development is a new type of multifunctional molecule based on the ANKET technology (named ANKET4), which incorporates an IL-2 engineered to promote specifically activation and proliferation of NK cells through the *cis*-engagement of NKp46, CD16a, and IL-2Rβ, without binding to IL-2Rα in Tregs or endothelial cells. In NHP, a CD20-targeting ANKET4 resulted in sustained B cell depletion with minimal systemic cytokine release [127].

A different platform for the development of multifunctional NKCE has been termed TriNKET (Trispecific NKCE Therapies), although not much information is available. Four molecules developed with this platform are being investigated in phase 1 trials, two of them for the treatment of hematological malignancies, both designed to simultaneously target CD16a, NKG2D and one TAA. CC-96191 is a CD33-targeting TriNKET in study in patients with AML (NCT04789655), while the BCMA-targeting CC-92328 is recruiting MM patients (NCT04975399).

#### IL-15-based trifunctional NK cells engagers

TriKE (Trispecific Killer cell Engagers) are trifunctional single-chain molecules that bind to a TAA with one arm, CD16 with the other and include IL-15 to promote activation of the recruited NK cells. GTB-3550 is fusion protein comprising two anti-CD16 and anti-CD33 scFv flanking human IL-15, which showed increased efficacy in vivo in an AML model compared to the corresponding BiKE without IL-15, and prolonged persistence of human NK cells [128]. Although a phase 1 clinical trial with GTB-3550 in AML patients (NCT03214666) showed expansion of endogenous NK cells and dose-dependent blast elimination [129], GTB-3550 was substituted by a second-generation TriKE (GTB-3650) with a humanized anti-CD16 VHH, more potent in preclinical studies [130]. Recently, it has been reported that the anti-CD33 TriKE GTB-3650 enhanced the activity of a CAR-NK therapy against AML cells in vitro [131]. GTB-5550 is a last generation TriKE with two VHH targeting CD16 on NK cells and B7H3 (CD276) expressed by multiple solid tumors and MM [132]. The B7-H3 TriKE promoted MM killing by NK cells through direct targeting and eliminated suppressive Mo-MDSC [133]. Manufacturing of GTB-5550 and GTB-3650 is underway, and phase I trials are expected in late 2023.

## Challenges and future perspectives

### Prevention of treatment-related adverse events

The primary toxicities following T cell-engaging immunotherapy administration are CRS and immune effector cell-associated neurotoxicity syndrome (ICANS). Several strategies have been used to prevent or minimize the severity of CRS due to T cell overactivation, including slower i.v. infusion or s.c. administration, adoption of step-up dosing (where patients are given a small, priming dose followed by an intermediate one, before receiving the full dose of the BsAb), the use of prophylactic corticosteroids, blockade of myeloid- or T cell derived cytokines (for example, with the anti-IL-6R mAb tocilizumab or the anti-TNF etanercept), pretreatment with a single-dose of the anti-CD20 mAb obinutuzumab (as de-bulking circulating B cells may dampen T cell activation), reduction of CD3 binding affinity (not yet been clinically validated), and the use of tyrosine kinase inhibitors as an emerging mitigation strategy [134].

On-target off-tumor toxicity in B cell hematological cancer patients treated with TCE is reasonably acceptable, since depletion of normal B or plasma cells is not life-threatening (in principle) and these cells are replenished after treatment. If necessary, hypogammaglobulinemia can be easily corrected with i.v. immunoglobulin replacement therapy. However, if the target antigen is present in normal tissues of vital organs, treatment may lead to fatal toxicity due to on-target, off-tumor effects, limiting considerably the therapeutic index of TCE in solid tumors [135–137]. Although not in the scope of this review, we would like to mention that the concept of conditionally active TCE is an area of considerable interest with potential implications for hematological malignancies. For instance, several engineered TCE constructs with masking domains designed to be specifically activated by proteases in the tumor microenvironment have demonstrated their potential for inducing protease-dependent cytotoxic activity with limited toxicity and are currently in clinical trials [138, 139]. Of note, other conditionally active TCE are designed for prolonged release, which could benefit a wider range of patients. One example is the TriTAC-XR, an extended-release TCE platform designed to minimize CRS by reducing maximum drug concentration in systemic circulation [140].

### T cell engagers and CAR-T cells: where do we stand?

CAR-T cell therapy represents a major breakthrough, limited, however, by individualized manufacturing timelines, whereas BsAbs are readily available off-the-shelf products. Regarding their safety profile, both therapeutic strategies have similar toxicities: CRS and immune effector cell-associated neurotoxicity syndrome (ICANS) [11]. Neurotoxicity in CD19-directed immunotherapies is similar, with grade ≥ 3 adverse events in at least 9–10% patients receiving blinatumomab or treated with CD19 CAR-T cells [141]. This has recently been attributed to the targeting of CD19-expressing pericytes, leading to BBB leakage [142]**.** In fact, studies with anti-CD20 TCE only report 0–4% grade ≥ 3 ICANS and 0–2% in patients treated with anti-BCMA TCE [143]. Regarding CRS, severe events (grade ≥ 3) were observed in 0–6% patients treated with blinatumomab [11], in 0–7% of patients receiving anti-CD20 TCE and in 0–4% of those treated with anti-BCMA TCE [143]. However, these percentages were three or more times higher is some studies involving patients treated with CD19 CAR-T cells or BCMA CAR-T cells [144]. Although comparisons among trials are intrinsically difficult and are further complicated by the use of different grading systems, the overall safety profile appears to be better for TCE BsAb molecules than for CAR T cells [145].

The key question is whether TCE can have a better safety profile and at least similar efficacy than CAR-T cells. Indeed, first generation TCE (exemplified by blinatumomab) were less potent than CAR-T cells (43% CR in patients with R/R B-ALL vs 81% CR in tisagenlecleucel-treated patients). However, the new generation of IgG-like TCE, with enhanced PK, may challenge the efficacy of CAR-T cells [146], although a proper assessment will require extended data on the duration of responses and whether cures can be achieved, particularly in patients with aggressive NHL. With CAR-T cell therapy, long-term data suggest that responses are durable, particularly for patients with CR. For example, the therapeutic potential of axicabtagene ciloleucel in patients with LBCL is supported by the 5-years follow-up of ZUMA-1 trial, but such long-term follow-up is still lacking for most TCE BsAb.

Finally, both treatment modalities are associated with substantial financial costs. Taking into account that blinatumomab achieves less CR than CAR-T cells (tisagenlecleucel), although its cost is considerably lower, cost-effectiveness analyses favor CAR-T cells when using blinatumomab as a comparator on parameters such as incremental QALYs (quality-adjusted life years) [147, 148]. However, as second generation TCE approach the clinical outcomes of CAR-T cells, they could compare favorably to CAR T cells once the costs of production, logistics, treatment, days of hospitalization, and short- and long-term adverse events have been considered. Importantly, the long-term response rate to TCE and CAR-T cell therapy will be critical to compare the cost-effectiveness of these immunotherapies [145].

Lagging behind T cell redirection are those strategies focused on the recruitment of NK cells: NKCE and CAR-NK. Although still preliminary, early reports on CAR-NK cells clinical trials in hematological malignancies suggest a better safety profile than CAR-T cells (neither CRS nor ICANS observed) [149]*.* Of note, the choice of NK cell source (for example, umbilical cord blood and induced pluripotent stem cells) may allow CAR-NK cells being considered off-the-shelf therapies. On the other hand, this strategy faces major hurdles regarding NK cell expansion and transduction efficiency. Indeed, both CAR-NK cells and second-generation NKCE candidates include IL15 to enhance NK proliferation and survival [107, 130].

### Alternative delivery methods for T cell engagers

Because of some of the above-mentioned limitations, investigators are exploring genetic approaches to produce BsAbs in vivo [150]. The endogenous secretion of TCE antibodies (STAb) by engineered cells [151] may have several advantages with respect to systemic administration of purified TCE. The in vivo secretion might result in effective concentrations of TCE compensating for the rapid renal filtration of small-sized TCE and avoid potential concerns regarding production and purification of antibodies. When compared to CAR-T cell therapy, the polyclonal recruitment by TCE of both engineered STAb-T cells and unmodified bystander T cells, present at the tumor site, might lead to a significant boost in antitumor T cell responses. The SATb-T strategy has demonstrated its therapeutic potential at the preclinical level in B cell and T cell malignancies using T cells secreting CD19 × CD3 or CD1a × CD3 BsAb, respectively [152, 153], and it could be further tailored to produce BsAb or TsAb formats for other indications.

## Conclusions

In recent years, there have been remarkable improvements in the immunotherapy of hematologic malignancies that are transforming outcomes in patients with relapsed/refractory disease. This also raises new challenges, such as the prioritization between different therapeutic strategies against the same target (e.g., anti-BCMA TCE, CAR-T cells and ADC) or the selection of a specific TAA among several targetable proteins in a given hematologic cancer. Indeed, we should not think in terms of TCE vs CAR-T cells (or NKCE vs CAR-NK cells) but rather how to use these therapies in combination or sequentially for patients to get the maximum benefit from the treatment. Whatever the scenario, multispecific antibodies (bispecific and beyond) will likely change the treatment paradigm in the coming years.

## Data Availability

Not applicable.

## Abbreviations

ADA: Anti-drug antibodies
AE: Adverse event
ADC: Antibody drug conjugated
ADCC: Antibody-dependent cell-mediated cytotoxicity
ADCP: Antibody-dependent cellular phagocytosis
AML: Acute myeloid leukemia
ANKET: Antibody-based NK cell engager technology
APRIL: A proliferation inducing ligand
BAFF: B cell activating factor
B-ALL: Acute B lymphoblastic leukemia
B-CLL: B cell chronic lymphocytic leukemia
BCMA: B cell maturation antigen
sBCMA: Soluble form of BCMA
BCR: B cell receptor
BEAT: Bispecific engagement by antibodies based on the TCR
BiKE: Bispecific killer cell engager
BiTE: Bispecific T cell engager
BLA: Biologics license application
BsAb: Bispecific antibody
CAR: Chimeric antigen receptor
CDC: Complement-dependent cytotoxicity
cFAE: Controlled fab-arm exchange
CODV: Cross-over dual variable
CR: Complete response
CRS: Cytokine release syndrome
DART: Dual affinity retargeting
DLBCL: Diffuse large B cell lymphoma
DVD-Ig: Dual variable domain immunoglobulin
Fab: Fragment antigen binding
Fc: Fragment crystallizable
FcyR: Fc gamma receptor
FCRH5: Fc receptor homologs 5
FDA: Food and Drug Administration
FL: Follicular lymphoma
GPRC5D: G Protein-coupled receptor class C group 5 member D
HSA: Human serum albumin
HL: Hodgkin lymphoma
HSC: Hematopoietic stem cells
Hu: Fully human
Hz: Humanized
ICANS: Immune effector cell-associated neurotoxicity syndrome
ICE: Immune cell engager
IgG: Immunoglobulin G
i.v.: Intravenous
IS: Immunological synapse
KiH: Knob into hole
mAb: Monoclonal antibody
MDS: Myelodysplastic syndrome
MM: Multiple myeloma
MRD: Minimal residual disease
NHP: Non-human primates
NK: Natural killer
NKCE: Natural killer cell engager
NKG2D: Natural killer group 2 D
NKp30: Natural killer receptor p30
NKp46: Natural killer receptor p46
NHL: Non-Hodgkin lymphoma
ORR: Overall response rate
PK: Pharmacokinetic
ROCK: Redirected optimized cell killing
R/R: Relapsed/refractory
s.c.: Subcutaneous
sdAb: Single-domain antibody
scFv: Single-chain variable fragment
SLAMF7: Signaling lymphocytic activation molecule family 7
TAA: Tumor-associated antigen
T-ALL: T cell acute lymphoblastic leukemia
TandAb: Tandem diabody
TCB: T cell bispecific antibody
TCE: T cell engager
TCR: T cell receptor
TDB: T-cell-dependent bispecific antibody
TNFR: Tumor necrosis factor receptor
TriNKET: Trispecific NKCE therapies
TriTAC: Trispecific T cell activating construct
TsAb: Trispecific antibody
VHH: Variable domain from heavy-chain only antibodies from camelids
VNAR: Variable domain from heavy-chain only antibodies from cartilaginous fish
